# Anovulatory Patients Demonstrate a Sharp Decline in LH Levels upon GnRH Antagonist Administration during IVF Cycles

**DOI:** 10.5041/RMMJ.10298

**Published:** 2017-04-28

**Authors:** Linoy Segal, Ofer Fainaru, Shahar Kol

**Affiliations:** IVF Unit, Rambam Health Care Campus, Haifa, Israel; and Rappaport Faculty of Medicine, Technion–Israel Institute of Technology, Haifa, Israel

**Keywords:** Estradiol pretreatment, GnRH antagonist, IVF, luteinizing hormone

## Abstract

To evaluate the decrease in luteinizing hormone (LH) levels following gonadotropin-releasing hormone (GnRH) antagonist administration in *in vitro* fertilization (IVF) cycles, data were retrospectively collected from 305 consecutive IVF or intracytoplasmic sperm injection (ICSI) cycles of patients who underwent ovarian stimulation with gonadotropins and were treated with GnRH antagonist for the prevention of premature luteinization. We compared the percent change in LH concentration from stimulation start to that observed before ovulation triggering in patients with or without anovulation. Anovulatory patients were younger, with higher body mass index (BMI), and demonstrated higher ovarian reserve parameters as compared to ovulatory patients. The decline in LH concentration was almost two-fold greater in anovulatory versus ovulatory patients. Numbers of oocytes, fertilizations, cleavage stage embryos, and transferred embryos were similar; however, implantation rates were higher in anovulatory versus ovulatory patients. Older patients (age ≥39) showed a smaller decline in LH levels as compared to younger ones (age <39) and exhibited poor IVF outcomes. There is a wide range of pituitary responses to GnRH antagonists. Anovulatory patients are more susceptible to GnRH antagonists and therefore demonstrate over-suppression of the pituitary. Older patients demonstrate a reduced pituitary response to GnRH antagonists than younger ones. Cycle scheduling with estradiol pretreatment did not influence LH decline, nor IVF treatment outcomes.

## INTRODUCTION

Gonadotropin-releasing hormone (GnRH) antagonists have been used with success in *in vitro* fertilization (IVF) clinics as of the late 1990s, but the ideal luteinizing hormone (LH) serum level in patients undergoing IVF is still under debate. Kolibianakis et al. provided evidence that a higher level of LH during the early follicular phase of GnRH antagonist cycles is related to a reduced chance of pregnancy.^1^ Nevertheless, low LH levels in the late follicular phase in IVF cycles are associated with lower fertilization rates and higher biochemical pregnancy rates.^2^

In the study by the Ganirelix dose finding study group, very low LH levels were achieved in the two highest-dose groups (1 and 2 mg), the implantation rates were significantly lower, and the miscarriage rates during the first 6 weeks after embryo transfer were relatively higher, compared to the lower doses (such as 0.25 mg daily dose).^3^ Early pregnancy loss in LH-suppressed subjects was also demonstrated in normo-gonadotropic women treated with GnRH agonists.^4^ Huirne et al. showed the potential role of changes in LH levels during the cycle, rather than absolute levels. Specifically, in their study pregnancies were not achieved when the change in LH level was either too great or too small, paralleling excessive or insufficient suppression of LH secretion.^5^

The minimum effective dose of GnRH antagonist able to prevent a premature LH surge in controlled ovarian hyperstimulation (COS) cycles is a daily dose of 0.25 mg.^3^,^6^ Luteinizing hormone levels decline immediately after the start of antagonist administration, and this is often followed by a gradual increase later in the cycle, which depends significantly on the antagonist dose.^3^,^5^ Those findings were observed also in GnRH antagonist pharmacodynamics and pharmacokinetics studies.^7^,^8^

In a previous study we demonstrated that 26% of patients stimulated with recombinant follicle-stimulating hormone (FSH), receiving only 0.25 mg of GnRH antagonists, displayed “over-suppression” (LH level 24 hours after GnRH antagonist injection <50% of its pre-injection level), as if they were exposed to a higher dose of antagonist. These patients may benefit from LH supplementation.^9^

Given the great variability of responses to GnRH antagonists, the primary objective of this study was to evaluate the decline in LH levels following antagonist administration, and to examine this response in different subpopulations with common causes of infertility (i.e. anovulatory patients and patients at advanced age).

## MATERIAL AND METHODS

A retrospective study was performed, analyzing data from 305 consecutive IVF or intracytoplasmic sperm injection (ICSI) cycles performed at the IVF Unit, Rambam Health Care Campus (Haifa, Israel) during the year 2015. All cycles performed at the unit that met the inclusion criteria were included in this study.

### Inclusion Criteria

We included IVF cycles of patients who underwent ovarian stimulation with gonadotropins and were administered a GnRH antagonist, Cetrotide 0.25 mg (Merck KGaA, Darmstadt, Germany), for the prevention of premature ovulation.

### Exclusion Criteria

Our study excluded patients who were administered a GnRH agonist for the prevention of premature ovulation, patients with a concurrent medical condition that could interfere with pregnancy outcome, and cycles where all available embryos were cryopreserved.

### Protocol

Ovarian stimulation was started on day 2 or 3 of a spontaneous menstrual period. A total of 90.5% of patients received both recombinant FSH (Gonal-f®, Merck KGaA, Darmstadt, Germany; Elonva®, Merck Sharp & Dohme Limited, Hoddesdon, Hertfordshire, UK) and LH (Pergoveris®, Merck KGaA, Darmstadt, Germany; Menopur® Ferring Pharmaceuticals, Parsippany, NJ, USA); 9.5% of cycles received only recombinant FSH. We administered GnRH antagonist (0.25 mg) once a lead follicle of 14 mm mean diameter was observed by ultrasound imaging. Recombinant human chorionic gonadotropin (hCG) (Ovitrelle 250 μg, Merck) or GnRH agonist (Decapeptyl 0.2 mg, Ferring) was administered once three leading follicles reached ≥17 mm mean diameter; oocyte retrieval was performed 34–36 h later. Oocytes were fertilized with conventional IVF or ICSI.

The primary end-point of the study was the percentage of decline in serum LH levels following GnRH antagonist administration (calculated as the percent change in LH concentration from stimulation start to that observed before ovulation triggering). The secondary end-points were: examination of the response to GnRH antagonist administration in different subpopulations (ovulatory, anovulatory, and advanced age) and IVF outcome parameters. The study was approved by the local institutional review board.

### Statistical Analysis

The comparisons of continuous data were performed using independent samples *t* test, and categorical data were compared using Pearson’s chi-square and Fisher’s exact tests, where appropriate. Significance was set at *P<*0.05 for all tests. Data are presented as mean±SEM. Statistical analysis was conducted using the Statistical Package for Social Sciences (SPSS version 20.0.0.0, IBM Corp., USA) and WINPEPI programs (J.H. Abramson, 2011, version 11.10, Jerusalem, Israel [http://bit.ly/2pjuuSC]).

## RESULTS

In total, 305 cycles of IVF or ICSI were included in the analysis. Cycle scheduling with estradiol 2 mg b.i.d. prior to gonadotropin administration was used in 191 cycles. We compared the primary and secondary outcomes in 269 cycles of ovulatory patients and 36 cycles of anovulatory patients (World Health Organization [WHO] group II).

Anovulatory patients were younger (28.3±5.6 versus 34.3±6.5 years; *P*<0.001), with higher BMI (27.3±6.0 versus 24.5±4.9 kg/m^2^; *P*=0.003), and demonstrated higher antral follicular counts (AFC) (19.9±10.3 versus 9.9±5.5; *P*<0.001) and lower basal FSH (5.3±1.5 versus 7.2±2.8 IU/L; *P*<0.001) as compared to ovulatory patients (Table 1).

**Table 1:** Baseline Characteristics in the Groups of Ovulatory and Anovulatory Patients.

	Ovulatory (*n*=269)	Anovulatory (*n*=36)	*P* Value
Age (years)	34.3±6.5	28.3±5.6	<0.001
BMI (kg/m^2^)	24.5±4.9	27.3±6.0	0.003
Etiology of infertility (%)			<0.001
Male factor	38.1	20.0	
Female factor	18.8	20.0	
Unexplained	37.3	20.0	
Combined	5.0	40.0	
Preimplantation genetic diagnosis	0.8	0.0	
Antral follicular count	9.9±5.5	19.9±10.3	<0.001
Basal FSH (IU/L)	7.2±2.8	5.3±1.5	<0.001

Cycle scheduling with estradiol pretreatment was performed in 55.6% of anovulatory patients, as opposed to only 34.9% of ovulatory patients (*P*=0.016, Table 2).

**Table 2:** Ovarian Stimulation Parameters in the Groups of Ovulatory and Anovulatory Patients.

	Ovulatory (*n*=269)	Anovulatory (*n*=36)	*P* Value
Estradiol pretreatment (%)			0.016
Yes	34.9	55.6	
No	65.1	44.4	
LH levels on stimulation start (IU/L)	6.2±4.1	6.8±4.4	0.42
Progesterone levels on stimulation start (nmol/L)	1.7±1.1	1.7±0.8	0.89
Estradiol levels on stimulation start (pmol/L)	307.8±225.6	269.3±209.5	0.33
Total FSH dose (IU)	1998.8±791.4	1602.4±522.8	0.004
LH levels on before ovulation triggering (IU/L)	3.0±2.8	1.8±1.2	0.02
Progesterone levels before ovulation triggering (nmol/L)	2.4±2.2	2.2±1.0	0.54
Estradiol levels before ovulation triggering (pmol/L)	5546.6±4107.8	6046.8±3975.0	0.49
Decline in LH levels from stimulation start to that before ovulation triggering (%)	38.9±64.9	66.4±26.3	0.01
Estradiol increment per oocyte (pmol/L)	938.7±1693.8	839.7±630.5	0.73

Hormone levels (LH, progesterone, and estradiol) on the day of stimulation start were similar between the two groups, although LH levels before ovulation triggering were lower in anovulatory patients (1.8±1.2 versus 3.0±2.8 IU/L; *P*=0.02). Therefore, the decline in LH concentration from stimulation start to that observed before ovulation triggering was almost two-fold greater in anovulatory versus ovulatory patients (66.4%±26.3% versus 38.9%±64.9%; *P*=0.01) (Table 2).

The total FSH dose was significantly lower in the anovulatory group (*P*=0.004) (Table 2).

Number of oocytes, fertilizations, cleavage stage embryos, and transferred embryos were all similar in the ovulatory and anovulatory patients. Implantation rates (27.8%±42.2% versus 11.5%±28.5%; *P*= 0.003) were higher in anovulatory versus ovulatory patients (Table 3).

**Table 3:** IVF Treatment Outcome in the Groups of Ovulatory and Anovulatory Patients.

	Ovulatory (*n*=269)	Anovulatory (*n*=36)	*P* Value
Number of oocytes retrieved	7.7±5.6	8.7±5.8	0.31
Number of fertilizations	4.3±3.6	4.7±4.4	0.64
Number of embryos obtained	2.8±2.2	3.2±3.1	0.29
Number of embryos transferred	1.7±0.8	1.5±0.8	0.19
Implantation rate (%)	11.4±28.5	27.8±42.2	0.003

Cycle scheduling with estradiol pretreatment led to increased LH levels on the day of stimulation start (6.7±4.5 versus 5.6±3.4 IU/L; *P*=0.03). Progesterone levels were significantly lower (1.6±1.1 versus 1.9±0.9 nmol/L; *P*=0.003), and estradiol was more than two-fold greater in the group pretreated with estradiol (Table 4). However, the decline in LH concentration from stimulation start to that observed before ovulation triggering was similar (42.8%±52.1 versus 42.9%±76.5; *P*=0.88), as were all other ovarian stimulation parameters (Table 4).

**Table 4:** Ovarian Stimulation Parameters in the Groups with or without Estradiol Pretreatment.

	With Estradiol Pretreatment (*n*=191)	Without Estradiol Pretreatment (*n*=114)	*P* Value
LH levels on stimulation start (IU/L)	6.7±4.5	5.6±3.4	0.03
Progesterone levels on stimulation start (nmol/L)	1.6±1.1	1.9±0.9	0.003
Estradiol levels on stimulation start (pmol/L)	390.1±240.6	159.1±62.3	<0.001
Total FSH dose (IU)	1969.7±777.4	1922.4±772.3	0.61
LH levels before ovulation triggering (IU/L)	3.0±2.8	2.7±3.2	0.45
Progesterone levels before ovulation triggering (nmol/L)	2.5±2.6	2.3±1.2	0.42
Estradiol levels before ovulation triggering (pmol/L)	5651.0±4160.8	5530.6±3983.6	0.84
Decline in LH levels from stimulation start to that before ovulation triggering (%)	41.8±52.2	42.9±76.5	0.88
Estradiol increment per oocyte (pmol/L)	994.7±1970.2	813.2±608.5	0.35

Number of oocytes, fertilizations, cleavage stage embryos, and transferred embryos and implantation rates were all similar in the groups with or without estradiol pretreatment (Table 5).

**Table 5:** IVF Treatment Outcome in the Groups with or without Estradiol Pretreatment.

	With Estradiol Pretreatment (*n*=191)	Without Estradiol Pretreatment (*n*=114)	*P* Value
Number of oocytes retrieved	7.6±5.4	8.2±5.9	0.43
Number of fertilizations	4.4±3.6	4.3±3.8	0.91
Number of embryos obtained	2.8±2.3	2.9±2.3	0.86
Number of embryos transferred	1.6±0.8	1.6±0.8	0.86
Implantation rate (%)	14.3±32.2	11.7±28.3	0.47

Older patients (age ≥39) showed a lower decline in LH levels from stimulation start to that before ovulation triggering (28.6%±79.2% versus 48.8%± 50.9%; *P*=0.008) in comparison to younger ones (age <39) (Table 6).

**Table 6:** Ovarian Stimulation Parameters in the Groups: Age ≥39 and Age <39.

	Age ≥39 (*n*=98)	Age <39 (*n*=207)	*P* Value
LH levels on stimulation start (IU/L)	5.8±3.5	6.5±4.4	0.13
Progesterone levels on stimulation start (nmol/L)	1.6±0.9	1.8±1.1	0.19
Estradiol levels on stimulation start (pmol/L)	287.3±220.0	310.8±225.71	0.39
Total FSH dose (IU)	2192.5±844.1	1838.2±713.7	<0.001
LH levels before ovulation triggering (IU/L)	3.4±3.1	2.6±2.5	0.03
Progesterone levels before ovulation triggering (nmol/L)	2.2±1.4	2.5±2.4	0.23
Estradiol levels before ovulation triggering (pmol/L)	4718.4±3585.2	6028.0±4251.6	0.009
Decline in LH levels from stimulation start to that before ovulation triggering (%)	28.6±79.2	48.8±50.9	0.008
Estradiol increment per oocyte (pmol/L)	1242.0±2680.8	782.1±639.4	0.02

Older patients (age ≥39) had fewer oocytes, fertilizations, and cleavage stage embryos (*P*=0.001), and a lower implantation rate (7.82%± 21.8% versus 15.9%± 33.9%; *P*=0.03) versus younger patients (Table 7).

**Table 7:** IVF Treatment Outcome in the Groups: Age ≥39 and Age <39.

	Age ≥39 (*n*=98)	Age <39 (*n*=207)	*P* Value
Number of oocytes retrieved	5.59±4.2	8.9±5.9	<0.001
Number of fertilizations	3.3±2.9	4.9±3.9	0.001
Number of embryos obtained	2.19±1.7	3.1±2.5	0.001
Number of embryos transferred	1.7±1.0	1.6±0.7	0.66
Implantation rate (%)	7.82±21.8	15.9±33.9	0.03

To examine the influence of the decline in LH levels from stimulation start to that before ovulation triggering we divided the data into two groups: if the LH level before ovulation triggering was less than 50% of the level measured on stimulation start, the subject was defined as “over-suppressed.” If the LH level was ≥50% was defined as “normal suppressed.”

The over-suppressed patients had a higher number of oocytes (8.4±5.7 versus 6.9±5.1; *P*=0.02), fertilizations (4.8±3.9 versus 3.7±3.0; *P*=0.01), and cleavage stage embryos (3.1±2.6 versus 2.4±1.9; *P*=0.004); however, implantation rates were similar (13.4%±30.4% versus 13.0%±31.1%; *P*=0.91). (Table 8).

**Table 8:** IVF Treatment Outcome in the Groups of Normal Suppressed and Over-suppressed.*

	Normal Suppressed (*n*=130)	Over-suppressed (*n*=175)	*P* Value
Number of oocytes retrieved	6.9±5.1	8.4±5.7	0.02
Number of fertilizations	3.7±3.0	4.8±3.9	0.01
Number of embryos obtained	2.4±1.9	3.1±2.6	0.004
Number of embryos transferred	1.6±0.9	1.6±0.8	0.75
Implantation rate (%)	13.0±31.1	13.4±30.4	0.91

* If the LH level before ovulation triggering was less than 50% of the level measured on stimulation start, the subject was defined as “over-suppressed.” If the LH level was ≥50% the subject was defined as “normal suppressed.”

Type of gonadotropin used (with or without LH activity) had no effect on LH suppression.

To assess the effect of elevation in LH levels from stimulation start to that before ovulation triggering we compared the IVF outcomes between patients that demonstrated an increase in LH levels to patients with decreased LH levels. The mean age of the two groups was similar. The patients with decreased LH levels showed a higher number of oocytes retrieved (8.1±5.6 versus 6.2± 5.5; *P*=0.05) and of embryos obtained (3.0±2.4 versus 2.0±1.7; *P*=0.01), but no significant difference in implantation rates (13.7%±30.9% versus 10.7%±30.3%; *P*=0.55) (Table 9).

**Table 9:** IVF Treatment Outcome in the Groups with Decline or Increase in LH Level from Stimulation Start to that before Ovulation Triggering.

	Decline in LH Level (*n*=263)	Increase in LH Level (*n*=42)	*P* Value
Age (years)	33.4±6.8	34.8±6.1	0.23
Number of oocytes retrieved	8.1±5.6	6.2±5.5	0.05
Number of fertilizations	4.5±3.6	4.0±3.8	0.44
Number of embryos obtained	3.0±2.4	2.0±1.7	0.01
Number of embryos transferred	1.7±0.8	1.5±1.0	0.16
Implantation rate (%)	13.7±30.9	10.7±30.3	0.55

## DISCUSSION

There is a wide variety in patients undergoing IVF treatment, both in basal characteristics, such as age and BMI, and in dynamic ones, such as their pituitary response to GnRH antagonists. This was demonstrated in our previous study where 26% of patients showed “over-suppression” by GnRH antagonist, while being stimulated with recombinant FSH. Pituitary over-suppression was associated with a low estradiol rise during ovarian stimulation, which was corrected by adding recombinant LH.^9^ It is well-established that a patient-individualized treatment approach can lead to the best obtainable clinical results in assisted reproductive technology.^10^–^12^ We therefore sought to examine the influence of over-suppressing endogenous LH levels on IVF treatment outcome, and to observe this effect in different subpopulations (anovulatory and advanced age patients) in order further to individualize our treatment approach.

In the current study, we now demonstrate that anovulatory patients are more susceptible to GnRH antagonist suppression as compared with ovulatory patients (Table 2). This effect cannot be attributed to estradiol-based scheduling (Table 2), because it does not affect pituitary response to GnRH antagonists (Table 4).

Notably, despite greater LH suppression, we observed higher implantation rates in anovulatory versus ovulatory patients (*P*=0.003, Table 3). This may have been attributed to the patients’ younger age (Table 1); however, when we compared these responders there was no influence of over-suppression on implantation rate (Table 8). Since 90.5% of included patients received both FSH and LH in their stimulation cycle, we suggest that in anovulatory patients LH suppression was corrected by exogenous supplementation. Therefore, LH activity-containing gonadotropins are probably preferable in antagonist cycles.

Older patients (age ≥39) showed a lower decline in LH levels from stimulation start to that before ovulation triggering (Table 6), indicating a reduced pituitary response to GnRH antagonists with increasing age. As expected, clinical outcome in the older group was inferior compared to younger patients (Table 7).

When assessing the effect of a blunted pituitary response to GnRH antagonists, we did note a lower number of oocytes retrieved when LH levels increased during stimulation, despite administration of a GnRH antagonist.

Our study limitation is mainly its retrospective design. Therefore, direct cause-and-effect relationships between the measured parameters cannot be fully established and should be validated in a prospective manner.

In conclusion, our study demonstrates that there is a wide range of pituitary responses to GnRH antagonists. Anovulatory patients (WHO group II) are more susceptible to GnRH antagonists and therefore demonstrate over-suppression of the pituitary as compared to ovulatory patients. Furthermore, older patients demonstrate a reduced pituitary response to GnRH antagonists compared to younger ones. Finally, estradiol-based scheduling does not affect pituitary response to GnRH antagonists and does not influence IVF outcome. Our results also suggest that LH over-suppression can be corrected by adding exogenous LH.

## Abbreviations

BMI: body mass index
COS: controlled ovarian hyperstimulation
FSH: follicle-stimulating hormone
GnRH: gonadotropin-releasing hormone
hCG: human chorionic gonadotropin
ICSI: intracytoplasmic sperm injection
IVF: *in vitro* fertilization
LH: luteinizing hormone
